# Comparative evaluation of standardized imaging-guided contact selection for subthalamic deep brain stimulation in Parkinson’s disease: study protocol for a randomized double-blind crossover trial

**DOI:** 10.1186/s13063-025-09396-3

**Published:** 2025-12-30

**Authors:** G. A. Brandt, L. Piotrowsky, J. N. Petry-Schmelzer, C. van der Linden, C. Schedlich-Teufer, V. Visser-Vandewalle, T. A. Dembek, M. T. Barbe

**Affiliations:** 1 Department of Neurology, Faculty of Medicine and University Hospital Cologne, University of Cologne, Cologne, Germany; 2 Department of Stereotactic and Functional Neurosurgery, Faculty of Medicine and University Hospital Cologne, University of Cologne, Cologne, Germany; 3 Department of Neurology with Experimental Neurology, Charité - Universitätsmedizin Berlin, corporate member of Freie Universität Berlin and Humboldt Universität zu Berlin, Berlin, Germany

**Keywords:** Parkinson’s disease, Deep brain stimulation, Subthalamic nucleus, Imaging-guided programming, Clinical trials

## Abstract

**Background:**

Subthalamic deep brain stimulation (STN-DBS) effectively treats motor symptoms in appropriately selected patients with Parkinson’s disease, but individual responses vary. Despite modern directional leads allowing more precise stimulation, optimal contact selection strategies have not yet been standardized. This study compares a standardized imaging-guided contact selection protocol to conventional clinical programming.

**Methods:**

We designed a monocentric, randomized, double-blind crossover trial enrolling 30 people with Parkinson’s disease with bilateral directional STN-DBS. Participants will undergo both programming approaches: standardized imaging-guided contact selection targeting the dorsolateral STN and conventional contact selection through clinical test stimulations. Each configuration will be applied for 1 week. The primary outcome is patient preference after both treatments. Secondary outcomes include motor assessments (MDS-UPDRS III), accelerometric monitoring, and questionnaire-based quality of life measures (e.g., PDQ-39).

**Discussion:**

This study addresses a critical gap in standardization of imaging-guided DBS programming. By using patient preference as the primary outcome, we aim to capture clinically meaningful differences that may not be detected with traditional motor scales. The crossover design balances statistical power with clinical feasibility in specialized care settings.

**Trial registration:**

Deutsches Register für Klinische Studien DRKS00034229. Registered on May 27, 2024.

## Administrative information

Note: the numbers in curly brackets in this protocol refer to SPIRIT checklist item numbers. The order of the items has been modified to group similar items (see http://www.equator-network.org/reporting-guidelines/spirit-2013-statement-defining-standard-protocol-items-for-clinical-trials/).

**Table:** 

Title {1}	Comparative evaluation of standardized imaging-guided contact selection for subthalamic deep brain stimulation in Parkinson’s disease: study protocol for a randomized double-blind crossover trial
Trial registration {2a and 2b}	Deutsches Register für Klinische Studien (DRKS00034229, May 27, 2024)
Protocol version	CONECT Prüfplan 1.2 (April 5, 2025)
Funding {4}	This study is conducted with internal institutional resources. No external funding was received.
Author details {5a}	Brandt GA^1,3^, Piotrowsky L^1^, Petry-Schmelzer JN^1^, van der Linden C^1^, Schedlich-Teufer C^1^, Visser-Vandewalle V^2^, Dembek TA^1^, Barbe MT^1^ ^1^University of Cologne, Faculty of Medicine and University Hospital Cologne, Department of Neurology ^2^University of Cologne, Faculty of Medicine and University Hospital Cologne, Department of Stereotactic and Functional Neurosurgery ^3^Charité - Universitätsmedizin Berlin, corporate member of Freie Universität Berlin and Humboldt Universität zu Berlin, Department of Neurology with Experimental Neurology
Name and contact information for the trial sponsor {5b}	Universität zu Köln Albertus-Magnus-Platz 50923 Köln D - Germany
Role of sponsor {5c}	No external entity had any role in study design, data collection, data analysis, data interpretation, writing of the report, or the decision to submit the manuscript for publication. All decisions related to the research process remained solely with the investigator team, with no external authoritative oversight or influence.

## Introduction

### Background and rationale {6a}

Subthalamic deep brain stimulation (DBS) is a well-established neuromodulation therapy used to alleviate the motor symptoms of Parkinson’s disease (PD) [1, 2]. It effectively reduces motor fluctuations and decreases the required dosages of dopaminergic medication, maintaining these benefits well beyond the immediate postoperative phase [3]. However, individual treatment responses remain variable, highlighting the need for further optimization to enable consistent clinical outcomes across a broader patient population [4].

A critical prerequisite for achieving optimal stimulation effects is the precise placement of the electric field [5, 6]. Extensive research has established that stimulation of the posterodorsal aspect of the subthalamic nucleus (STN) is most effective in reducing akinetic-rigid symptoms with minimal side effects [7–10]. To further enhance the precision of stimulation beyond the initial implantation, modern DBS leads are equipped with multiple segmented contacts [11, 12]. These allow for vertical and horizontal current steering, thereby facilitating more targeted stimulation [13].

Traditionally, optimal stimulation contacts are identified through a monopolar review, i.e., multiple test stimulations conducted during a comprehensive clinical examination by a DBS expert [14, 15]. While generally deemed effective, this process is cumbersome, subjective, and time-consuming and has become increasingly more complex with the modern DBS lead design [16, 17].

In recent years, several software solutions have been introduced that enable visualization of the individual lead position in relation to the target brain structures [18, 19]. Multiple studies have demonstrated that expert clinicians can determine effective stimulation contacts using this imaging data in a more time-efficient manner compared to conventional approaches [20–23]. However, the optimal strategy for imaging-guided DBS programming remains to be established. Comparative correlation analyses suggest that even minor differences in contact selection strategies may significantly impact treatment outcomes [24].

So far, efforts to standardize imaging-guided DBS programming have been limited. Prior investigations have refrained from specifying the exact algorithms or decision-making processes used for contact selection based on visual information, thereby limiting reproducibility and generalizability. To address this gap, we designed a randomized double-blind crossover trial to compare a standardized protocol for imaging-guided contact selection to conventional DBS programming.

### Objectives {7}

Does standardized imaging-guided contact selection facilitate equal symptom control compared to conventional contact selection for STN-DBS in PD?

### Trial design {8}

This study is a randomized, controlled, double-blind, crossover trial with an intra-individual head-to-head comparison of two deep brain stimulation (DBS) programs. The allocation ratio is 1:1, with each participant receiving both interventions in a randomized sequence. The trial follows an equivalence framework, with the primary outcome being tested for equality using the Prescott test.

The Prescott test was selected specifically because it detects period-by-treatment interactions while testing for preference. A washout period was not included as DBS effects typically subside within minutes after reprogramming, unlike pharmacological interventions, and are often omitted in DBS trials [25, 26].

## Methods: participants, interventions, and outcomes

### Study setting {9}

This study will be executed as a monocentric study (University Hospital Cologne, Cologne, Germany).

### Eligibility criteria {10}

Inclusion criteria:

- Clinically confirmed diagnosis of Parkinson’s disease according to current MDS criteria [27]
- Implanted DBS system with bilateral directional leads for at least 10 weeks
- Availability of high-resolution cranial preoperative MRI and postoperative CT from routine clinical imaging
- Stable Parkinson’s disease-specific medication regimen for at least 2 weeks
- Age over 18 years

Exclusion criteria:

- Debilitating postural instability
- Cognitive impairment meeting diagnostic criteria for dementia
- Vestibular or orthopedic comorbidities with significant impact on daily functioning
- Dopamine dysregulation syndrome
- Inability or unwillingness to reliably operate the DBS system’s remote control

### Who will take informed consent? {26a}

Informed consent will be obtained from all potential participants both in written and oral form by study physicians prior to enrollment in the trial.

### Additional consent provisions for collection and use of participant data and biological specimens {26b}

No biological specimens will be collected in this study. Participant data will be collected according to established guidelines, with no provisions for use in ancillary studies. The informed consent process addresses only the data collection and use for the current trial.

## Interventions

### Explanation for the choice of comparators {6b}

The two DBS programming approaches were selected for comparison based on their clinical relevance and potential impact on patient care. Conventional clinical contact selection through test stimulations represents the current standard of care in most DBS centers worldwide and has established efficacy. However, this approach is time-consuming, requires specialized expertise, and may be subject to inter-examiner variability [20, 21].

Standardized imaging-guided contact selection has emerged as a potentially more efficient alternative that might provide more consistent outcomes across centers [23]. Our specific standardized protocol targeting the dorsolateral STN is based on our clinical experience with imaging-guided DBS programming and previous research showing this approach’s association with optimal motor improvement [24].

### Intervention description {11a}

The study intervention will be a DBS program with a contact selection based on individual imaging. The required imaging is acquired during our clinical routine: preoperative isometric (1.0 mm^3^ voxel size) T1- and T2-weighted MRI (3 T Ingenia, Achieva, 1.5 T Ingenia, Philips Healthcare, The Netherlands) and a postoperative high-resolution CT scan (IQon Spectral CT, iCT 256, Brilliance 256, Philips Healthcare, The Netherlands). Imaging will be assessed with a proprietary software (Brainlab Elements GUIDE XT, Brainlab, Munich, Germany). After automated fusion of the available images, three nuclei will be segmented using the integrated segmentation function (nucleus ruber, substantia nigra, subthalamic nucleus). The dorsal, lateral, and medial borders of the STN are refined based on the T2 hypointensity. The lead positions will be determined using the automated lead detection algorithm. Lead orientations will either be determined via the automatic lead orientation detection algorithm or assessed visually, if necessary. Standardized imaging-guided contact selections will be determined with the 3D visualization of a representative VTA (volume of tissue activated; 2 mA, 60 µs, 130 Hz), which is positioned following a predetermined strategy [24]:

1. If a lead is positioned in the visual center of the posterior third of the STN, omnidirectional settings are selected. Otherwise, the maximal directional focus is applied and faced toward the posterior third of the STN.
2. Contact levels close to the dorsal border of the STN are selected to align the dorsal border of the VTA with the dorsal border of the STN, focusing on the dorsal motor STN. If a ring contact is the closest to the dorsal border of the STN with a lead positioned off-center to the posterior third of the STN, directional contacts on the neighboring level can be used to facilitate directional stimulation at the investigator’s discretion.

The control intervention will be a DBS program with a contact selection determined during a physical examination after overnight cessation of dopaminergic medications with test stimulations by movement disorder experts with extensive experience in DBS treatment. During the examination, the most effective level will be determined; multi-level configurations are permitted at the clinician’s discretion. Directional settings will be selected, when the clinician deems a clinical benefit during test stimulations.

Both DBS programs will apply pulses with 60 µs at 130 Hz. The initial amplitudes are determined based on a subsequent blinded amplitude titration and determined as rigidity threshold + 0.5 mA. Each DBS program will be applied for a week. Study participants will be asked to autonomously titrate the optimal amplitudes during the first 3 days of the respective treatment weeks. During the following 3 days, amplitudes will be kept stable.

### Criteria for discontinuing or modifying allocated interventions {11b}

Adverse events, including stimulation-induced side effects, will be monitored and documented throughout the study duration. Participants retain the right to withdraw from the study at any time without explanation. Should a participant choose to discontinue a treatment week, they may still proceed to the subsequent treatment phase if the aborted week was their first allocation. Assessment of treatment preference will occur regardless of whether participants complete both treatment weeks. To ensure patient safety, each participant will have their established DBS program available as a rescue option accessible via the patient remote.

### Strategies to improve adherence to interventions {11c}

To ensure adherence to the intervention protocols, each participant will receive a detailed schedule of their treatment periods, instructions for indications for amplitude adjustments, and a motor diary to record daily experiences with each DBS program during the stable treatment phase. Study personnel will conduct regular phone check-ins between scheduled visits to monitor protocol adherence and address any concerns. During on-site visits, the research team will verify DBS program settings to confirm participants are receiving the allocated intervention.

### Relevant concomitant care permitted or prohibited during the trial {11d}

Participants will be advised to maintain their regular medication regimen and routine care throughout the trial period. Participants are prohibited from enrolling in other interventional clinical trials during the study period.

### Provisions for post-trial care {30}

Upon trial completion, participants will transition to regular care at our hospital. Any adverse events related to trial participation will be treated according to standard clinical protocols within our institution. Participants are covered by insurance coverage for trial-related injuries or harm.

### Outcomes {12}

The primary outcome will be patient preference between imaging-guided contact selection and clinical contact selection, which will be captured at the end of the second treatment week as a choice between week 1 and week 2. Participants are allowed to refrain from stating a preference. Secondary outcome measures include clinical (MDS-UPDRS III) and objective (accelerometer) motor symptom assessments as well as patient questionnaires (FOG-Q, PDQ-39, MDS-UPDRS I/II/IV, MDS-NMSS) and motor diaries. Additionally, clinical characteristics of the study population including age, disease duration, and current medication, as well as cognitive status (Montreal Cognitive Assessment), will be recorded at baseline.

### Participant timeline {13}

See Fig. 1.

**Fig. 1:**
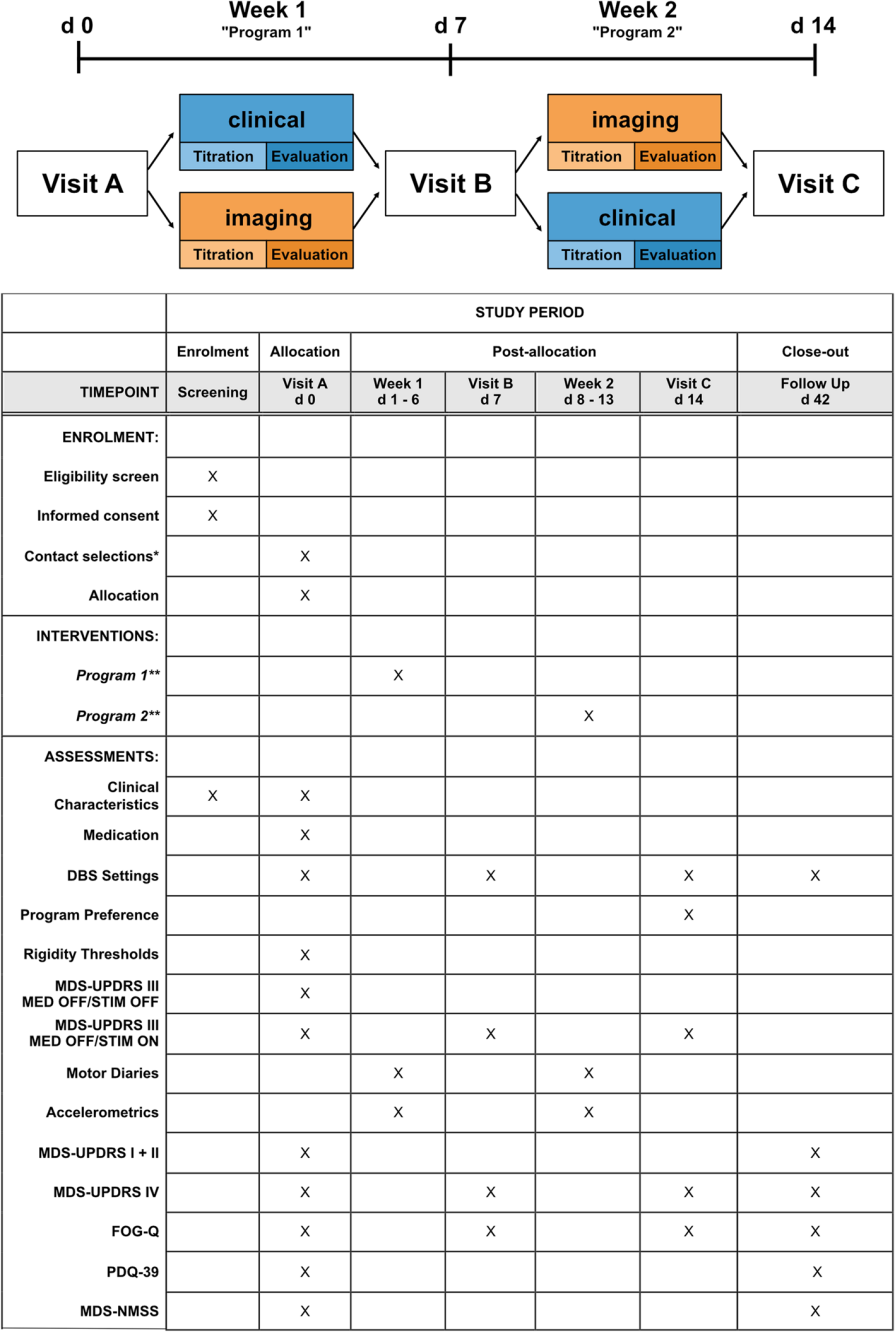
Schematic trial timeline including a schedule of enrollment, interventions, and assessments

### Sample size {14}

**Fig. 2:**
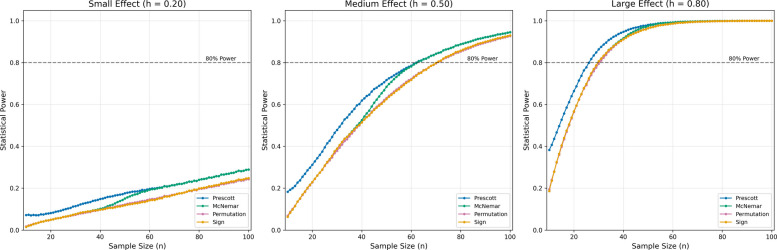
Statistical power by effect and sample size: Monte Carlo simulation with pseudorandomized probabilities for a binary choice (*p*(*b*) = 1 − *p*(*a*)) with four different test statistics. Each panel shows the analysis for probability distributions representing either a small (left), medium (middle), or large (right) treatment effect as defined by Cohen’s *h*. While permutation statistics (red) and the sign test (yellow) lead to more conservative power estimations across the spectrum of sample sizes than Prescott (blue) and McNemar (green) tests, below the sample size of 60 the Prescott test yields the highest power among the compared tests

Our comparative power simulation (Monte Carlo method with 100,000 iterations, *p*(*b*) = 1 − *p*(*a*), *n* = 10:1:100) evaluated four binary outcome tests across Cohen’s *h* effect sizes. Results revealed the Prescott test of equality minimizes false negatives in small cohorts (*n* < 60) compared to McNemar, sign test, and permutation statistics (Fig. 2). This specialized test was developed in 1981 specifically for crossover designs and demonstrates superior robustness against treatment order effects [26].

Based on the simulations within the framework of Cohen’s *h*, we determined that a study with 27 subjects would achieve 80% statistical power at *α* = 0.05 (two-sided) to detect a true preference difference for detecting large, i.e., substantial and clinically significant differences (Fig. 2). In the binary choice scenario of this study, Cohen’s *h* = 0.8 corresponds to a preference distribution of 70% versus 30%. Anticipating a dropout rate of 10%, we plan to recruit 30 subjects. This investigation will be sufficiently powered to detect or exclude large treatment effects. A non-significant result would provide evidence against the presence of a large treatment effect, though smaller clinically relevant differences may remain undetected and would require larger sample sizes to identify.

### Recruitment {15}

Study participants will be recruited from the patient population of the Neurological Department of the University Hospital Cologne. Suitable candidates will be prescreened based on the available medical records. For definitive screening, possible study participants will be reexamined by a study physician to assess inclusion and exclusion criteria.

## Assignment of interventions: allocation

### Sequence generation {16a}

The allocation sequence will be generated through block randomization. Blocks containing six tokens (3 × AB/3 × BA treatment sequences) will be prepared to ensure balanced allocation of treatment order.

### Concealment mechanism {16b}

The allocation sequence will be concealed using prepared envelopes containing the randomized treatment order tokens. The documented treatment order and interventions will be stored securely in sealed envelopes and a password-protected database.

### Implementation {16c}

One designated study team member will determine the treatment order by drawing from the prepared envelopes, establish the imaging-guided contact selection, and handle the programming devices. This team member will enroll participants and assign them to the intervention sequences. The remaining team members will remain blinded to patient anatomy and active treatment throughout the study.

## Assignment of interventions: blinding

### Who will be blinded {17a}

This study employs a double-blind design. Trial participants, clinical assessors, and all study team members except for one designated programmer will be blinded to the treatment allocation. The designated programmer will determine treatment order, establish the imaging-guided contact selection, and handle the programming devices, while maintaining confidentiality of the allocation. Clinical DBS programming will be conducted by blinded team members who have no access to the treatment allocation information. Motor scales will be assessed based on video documentation to facilitate blinded ratings.

### Procedure for unblinding if needed {17b}

Unblinding will only be permitted in the event of a serious adverse event (SAE) where knowledge of the active DBS program is essential for appropriate clinical management and patient safety. All instances of unblinding will be documented, including the reason and personnel involved.

## Data collection and management

### Plans for assessment and collection of outcomes {18a}

All outcome assessments will be performed by trained clinical investigators. To promote data quality, standardized clinical research forms (CRFs) will be used for all assessments, and any modifications will be clearly marked and signed by the investigator.

### Plans to promote participant retention and complete follow-up {18b}

To promote participant retention and complete follow-up, participants will receive clear scheduling information at enrollment and reminder calls prior to each study visit. The relatively short duration of each treatment phase (1 week per intervention) minimizes participant burden. Should a participant choose to discontinue a treatment week, they may still proceed to the subsequent treatment phase if the aborted week was their first allocation. For participants who discontinue, we will collect the primary outcome measure (treatment preference) whenever possible, regardless of whether they complete both treatment weeks. Secondary outcome measures will be collected at the time of discontinuation to allow for partial analysis. All participants who receive at least one intervention will be included in the safety analysis. Transportation support will be offered to participants who have difficulty attending follow-up visits to minimize attrition.

### Data management {19}

Data will be collected on physical clinical research forms (CRFs) with checklists and tables to ensure complete data entry. All CRF modifications will be clearly marked and signed by the investigator. The data will then be transferred to a dedicated spreadsheet for analysis. To ensure data quality, both source documentation and CRFs will be subject to monitoring by the Zentrum für Klinische Studien Köln (ZKS Köln). The monitors will verify 100% of inclusion and exclusion criteria as well as the primary outcome measure for all subjects. Additional random sampling of other data points will be conducted during regular monitoring visits to verify overall data integrity. The dedicated spreadsheet will be password-protected and regularly backed up to a designated secure hard drive within the hospital’s network.

### Confidentiality {27}

Personal information about participants will be protected through a coding system. Each participant will be assigned a unique anonymized code (e.g., CONECT01) that will be used on all study documentation and data. The master list linking participant identities to their assigned codes will be maintained separately from the study data in two secure locations: as an electronic file on the hospital’s secured computer system with restricted access and as a physical copy stored in the investigator site file kept in a locked cabinet with controlled access. Only authorized study personnel will have access to this decoding information.

### Plans for collection, laboratory evaluation, and storage of biological specimens for genetic or molecular analysis in this trial/future use {33}

Not applicable. This study does not involve the collection, evaluation, or storage of biological specimens.

## Statistical methods

### Statistical methods for primary and secondary outcomes {20a}

The binary outcome of patient preference will be coded as 0 (clinical contact selection) or 1 (imaging-guided contact selection) and analyzed with the Prescott test [28]. Based on the power simulations described above, the h0-hypothesis is defined as follows: “Based on patient preference, there is not any large, i.e., substantial and clinically significant difference between treatments (Cohen’s *h* > 0.8).” A *p* value < 0.05 indicates a clinically relevant difference between treatments.

The Prescott test assumes no carryover effects. Prior to the primary analysis, we will test for sequence effects by comparing preference rates between AB and BA groups. If significant carryover is detected (*p* < 0.10), only first-period data will be analyzed.

The secondary outcomes (MDS-UPDRS, PDQ-39, MDS-NMSS, FOG-Q, and motor diaries) will be compared across groups and timepoints using repeated measures ANOVA for normally distributed data or Kruskal-Wallis test for non-normally distributed data. Prior to analysis, data will be tested for normality using the Shapiro-Wilk test. The accelerometric data will be collected and kept for post hoc analysis.

### Interim analyses {21b}

For interim safety and efficacy evaluation, a formal analysis will be conducted after completion of the third randomization block (18 subjects, representing >50% of enrollment). The trial will be terminated early if either of two predefined stopping criteria are met:

i) Statistical significance (*p* < 0.05) of the Prescott test for the primary endpoint indicating a very large treatment effect.
ii) Treatment discontinuation exceeding 70% in either study arm indicating a safety signal.

### Methods for additional analyses (e.g., subgroup analyses) {20b}

Additional analyses will include both subgroup analyses and mixed modeling approaches. Clinical characteristics including disease type (akinetic-rigid, tremor-dominant, mixed presentation), disease duration, levodopa equivalent daily dose (LEDD), age, and cognitive status (MoCA score) will serve as independent variables. Composite subscores from assessment scales, such as dyskinesia and off-period scores from UPDRS IV, will also be analyzed. Mixed effects models will be employed to account for the repeated measures design and individual variability, with treatment condition as fixed effect and participant as random effect. Interaction terms between treatment condition and subgroup variables will be formally tested to assess whether treatment effects vary significantly across subgroups. These models will allow for examination of potential moderators of treatment effects.

Given that the sample size calculation was based on the overall study population rather than individual subgroups, these analyses will be underpowered to detect clinically meaningful differences within subgroups. These analyses will be considered exploratory and will be clearly identified as such in the reporting of results.

### Methods in analysis to handle protocol non-adherence and any statistical methods to handle missing data {20c}

The primary analysis will follow a modified intention-to-treat approach, including all randomized participants who received at least one intervention and stated a preference. For handling missing data, if a maximum of two components of a multiple component score (such as MDS-UPDRS III) is missing, the missing item value(s) will be imputed using the most frequent (mode) response to this item in the entire group at the corresponding time point. If a score is completely missing, the patient will be excluded from the main analysis of the corresponding endpoint. For the primary outcome of patient preference, if a participant fails to complete both treatment phases but provides a preference statement, this will be included in the analysis.

### Plans to give access to the full protocol, participant-level data, and statistical code {31c}

The full study protocol will be made available upon reasonable request to the corresponding author. Participant-level data may be shared for academic purposes following study completion, subject to appropriate data sharing agreements and ethical approvals.

## Oversight and monitoring

### Composition of the coordinating center and trial steering committee {5d}

This single-center study is conducted by a small study team consisting of the principal investigator and co-investigators who are responsible for day-to-day trial operations, including participant recruitment, intervention delivery, data collection, and management. The Zentrum für Klinische Studien Köln (ZKS Köln) provides institutional monitoring services and oversight to ensure compliance with Good Clinical Practice guidelines. The ZKS Köln conducts regular monitoring visits to verify source documentation, data integrity, and protocol adherence at the following milestones (after enrollment of >5 participants, after enrollment of 18 participants (interim analysis), and after the enrollment of the last participant). Additional visits may be scheduled based on risk assessment or if issues are identified. Monitoring findings will be documented in written reports provided to the principal investigator. There are no separate steering committee, endpoint adjudication committee, or external data management team for this trial.

### Composition of the data monitoring committee, its role and reporting structure {21a}

Due to the short duration of the study, the crossover design, and the low-risk profile of the interventions (comparing two established DBS programming approaches rather than testing a novel intervention), a formal Data Monitoring Committee has not been established for this trial. Both DBS programming approaches under investigation represent variations of standard clinical care, with well-characterized safety profiles. Safety monitoring will be conducted by the study team in conjunction with the Zentrum für Klinische Studien Köln (ZKS Köln), which provides independent monitoring services. Predefined stopping rules have been established for the interim analysis, and these decisions will be made by the principal investigator in consultation with the ZKS Köln based on the predefined criteria.

### Adverse event reporting and harms {22}

Adverse events (AEs) and serious adverse events (SAEs) will be collected, assessed, and documented throughout the study period. At each study visit, participants will be actively questioned about any experienced adverse events, which will be documented in the CRF. Spontaneously reported adverse events will also be documented accordingly. SAEs will be reported to the principal investigator within 24 h of detection and will be documented in detail, including onset, duration, resolution, and measures taken. SAEs that are related to the study intervention and unexpected will trigger the unblinding procedure and be reported to the Ethics Committee according to local requirements.

### Frequency and plans for auditing trial conduct {23}

No formal auditing procedures are planned for this trial beyond the regular monitoring provided by the Zentrum für Klinische Studien Köln (ZKS Köln). The monitoring activities conducted by ZKS Köln will verify adherence to the protocol, accuracy of data collection, and compliance with regulatory requirements. If concerns arise during monitoring, the principal investigator will implement corrective actions as needed. The institutional ethics committee retains the right to audit the trial at their discretion, which would be conducted independently from the investigators.

### Plans for communicating important protocol amendments to relevant parties (e.g., trial participants, ethical committees) {25}

Any modifications to the protocol that may impact the conduct of the study, participant safety, and potential benefit, or that significantly affect study procedures, objectives, or design will require a formal amendment. All amendments will be submitted to the Institutional Review Board/Ethics Committee for approval before implementation. Once approved, changes affecting participants will be communicated through written addenda to the informed consent form, with re-consent obtained when required by the ethics committee.

### Dissemination plans {31a}

Trial results will be disseminated through publication in peer-reviewed scientific journals and presentation at relevant scientific conferences, regardless of the outcome. There are no publication restrictions. The results will also be reported in the clinical trial registry where the study was registered. Participants will receive a summary of the study findings upon request.

This study protocol was written in accordance to the SPIRIT guidelines [29].

## Discussion

Due to the substantial clinical efficacy of subthalamic DBS, significant treatment effects can be reliably demonstrated using the motor exam of the long-established MDS-UPDRS [2, 30, 31]. While the strengths of this measure entail a broad validation, strong correlation with disease severity and established inter- and intra-rater variabilities [32, 33], the subtle but clinically relevant differences between two DBS programs, such as stimulation-induced dysarthria, can easily be underrepresented (1/132 points) in this measure [34–36]. To sensitively detect treatment differences both regarding treatment efficacy and side effects, we propose patient preference after a crossover trial as a meaningful measure of perceivable and clinically meaningful differences in treatment-related quality of life. The dichotomous nature of this endpoint offers statistical advantages over the inherent variability found in semi-quantitative assessment tools such as the MDS-UPDRS.

Patient preference was assessed using a single binary choice question. This approach was selected over complex preference instruments for several reasons: (1) it directly reflects the clinical decision faced by patients and physicians, (2) it maximizes statistical power in a small sample, (3) it allows patients to holistically integrate multiple outcome domains according to their individual values, and (4) it eliminates scaling biases inherent in graded preference measures.

To facilitate most valid comparison, the DBS programs will be compared by the study participants after 1 week of treatment with each program. One week allows stimulation effects to stabilize beyond initial adjustments and to be explored in the participants’ environment, while also being brief enough for recall.

As the expected difference in preference, i.e., the treatment effect cannot be determined a priori, we refrained from guessing expected distributions, but employed the well-established framework of Cohen’s *h* [37]. While a small treatment effect (*h* = 0.3) is considered measurable, but insignificant, a large treatment effect (*h* = 0.8) is considered substantial and clinically relevant. We conclude that while the detection of modest effects requires 63 subjects, a large difference in patient preference can be excluded (with a power > 80%) after a negative trial with 27 subjects.

While sufficiently powered for the primary outcome, the sample size of 30 participants limits the statistical power for subgroup analyses and the interpretation of non-significant findings in secondary outcomes. Additionally, generalizability may be restricted given the heterogeneous nature of Parkinson’s disease beyond a smaller study population.

### Perspective

The specialized nature of DBS care necessitates a multi-disciplinary team approach within tertiary centers, which traditionally constrains sample sizes [38]. This limitation stands in stark contrast to the rapidly expanding technological innovations in the field and the heterogeneous disease presentations, characterized by diverse clinical phenotypes and scenarios observed in everyday clinical practice. This dichotomy creates an urgent need for methodologically robust studies that are sufficiently powered yet practically feasible within existing clinical infrastructures.

The challenge facing the field is substantial: how to generate high-quality evidence that can withstand scientific scrutiny while acknowledging the practical constraints of clinical realities. Traditional clinical trial designs often fail to accommodate these tensions, resulting in either underpowered studies or impractical protocols that cannot be implemented in real-world settings.

Based on these considerations, we propose this specialized study design as a methodological bridge—combining statistical efficiency with clinical practicality. Our approach enables meaningful comparisons between DBS treatment strategies while remaining feasible within the constraints of specialized tertiary care settings. We recognize certain trade-offs in this approach: a smaller patient population may limit broader generalizability, and crossover designs carry inherent assumptions about treatment effects. Rare adverse events can go undetected before broader implementation and multi-center validation will be needed to verify study results.

The framework may also inform trial design beyond this specific comparison, enabling efficient investigation of other DBS innovations (adaptive versus conventional stimulation, dual-frequency versus single-frequency paradigms) and extending to other movement disorders where similar recruitment constraints exist, such as essential tremor and dystonia. The proposed design maximizes the information yield from necessarily limited sample sizes, potentially accelerating the translation and dissemination of technological advances into improved patient outcomes.

## Trial status

Protocol version: CONECT Prüfplan 1.2 (April 5, 2025). Recruitment began on July 10, 2024. The anticipated completion date for recruitment is March 31, 2026.

## Data Availability

The final trial dataset will be accessible to the principal investigator and co-investigators directly involved in the trial. There are no contractual agreements in place that limit access to the dataset for investigators. De-identified data may be made available to other researchers upon reasonable request following study completion, subject to appropriate data sharing agreements and ethical approvals. Statistical analyses will be performed by the study team, with no restrictions on investigator access to analyzed data.

## Abbreviations

DBS: Deep brain stimulation
STN: Subthalamic nucleus
STN-DBS: Subthalamic deep brain stimulation
PD: Parkinson’s disease
MDS-UPDRS: Movement Disorder Society-Unified Parkinson’s Disease Rating Scale
FOG-Q: Freezing of Gait Questionnaire
PDQ-39: Parkinson’s Disease Questionnaire-39
MDS-NMSS: Movement Disorder Society-Non-Motor Symptom Scale
CT: Computed tomography
MRI: Magnetic resonance imaging
VTA: Volume of tissue activated
LEDD: Levodopa equivalent daily dose
MoCA: Montreal Cognitive Assessment
SAE: Serious adverse event
AE: Adverse event
CRF: Clinical research form
ZKS Köln: Zentrum für Klinische Studien Köln (Center for Clinical Studies Cologne)
